# Contrasting edaphic and land-use signatures of microbial phosphorus mobilization and accumulation genes

**DOI:** 10.1093/ismejo/wrag272

**Published:** 2026-10-20

**Authors:** Mingming Du, Peipei Xue, Budiman Minasny, Yijia Tang, Thi Kim Anh Tran, Alex McBratney

**Affiliations:** School of Life and Environmental Sciences & Sydney Institute of Agriculture, The University of Sydney, Sydney, New South Wales 2015, Australia; School of Life and Environmental Sciences & Sydney Institute of Agriculture, The University of Sydney, Sydney, New South Wales 2015, Australia; School of Life and Environmental Sciences & Sydney Institute of Agriculture, The University of Sydney, Sydney, New South Wales 2015, Australia; School of Life and Environmental Sciences & Sydney Institute of Agriculture, The University of Sydney, Sydney, New South Wales 2015, Australia; School of Life and Environmental Sciences & Sydney Institute of Agriculture, The University of Sydney, Sydney, New South Wales 2015, Australia; School of Life and Environmental Sciences & Sydney Institute of Agriculture, The University of Sydney, Sydney, New South Wales 2015, Australia

**Keywords:** soil phosphorus cycling, land use, microbial biogeography

## Abstract

Phosphorus (P) is an essential nutrient that constrains productivity across many terrestrial ecosystems. Soil microorganisms regulate P transformation through distinct functional strategies, yet how these strategies vary with land use and environmental conditions across chronically P-impoverished landscapes remains poorly understood. Here, we combined 268 soil metagenomes from natural, pasture, and cropland systems across Australia with genome-resolved analyses of 1077 metagenome-assembled genomes. Results revealed contrasting genomic distributions among P-cycling strategies. P accumulation genes were widely distributed across bacterial phylogeny, whereas mineralization and solubilization genes were concentrated within specific lineages. Land-use-associated differences were most evident in the relative abundances of mineralization and solubilization genes, whereas accumulation genes showed comparatively little variation. Soil properties were stronger predictors of relative P-cycling gene abundance than climatic variables. Among these variables, soil pH was the strongest predictor of both the combined relative abundance of P-cycling genes and mineralization genes, whereas accumulation genes were negatively associated with the mineral associated organic carbon fraction. Continental predictions further suggested higher combined relative P-cycling gene abundance in wetter south-western and coastal eastern regions. These analyses reveal contrasting patterns among microbial P-cycling gene strategies across Australian soils and provide a genome-resolved framework for understanding their phylogenetic distributions and environmental associations at the continental scale.

## Introduction

Phosphorus (P) is a fundamental nutrient for biological growth [1]. In soils, P exists in multiple organic and inorganic pools, but plants mainly acquire it as inorganic orthophosphate [2]. Consequently, available P is frequently insufficient to meet plant demand, and ~43% of the natural terrestrial surface is considered relatively P-limited [3, 4]. This constraint is severe in Australia’s highly weathered soils, where total P concentrations are generally below the global average [5]. Under chronic P scarcity, soil microorganisms can influence P availability through distinct functional strategies, including organic P mineralization, mineral phosphate solubilization, and intracellular phosphate uptake and storage [6]. However, how these microbial P-cycling strategies are organized and reshaped in chronically P-poor systems remains poorly understood, despite their importance for sustainable P management under global environmental change.

The microbial phosphorus adaptive evolution framework emphasizes that chronic P scarcity selects for greater investment in P acquisition and accumulation [7]. Microbial trait theory further predicts that low complexity traits are widely dispersed across taxa, whereas complex multi-gene traits are more phylogenetically conserved [8]. Global surveys have shown that human activities shift the abundance of soil P-cycling genes [9, 10]. However, community-level gene abundance cannot link co-occurring genes to individual organisms and therefore cannot reveal how different P strategies are combined within a single genome. Genome-resolved approaches can address this gap by linking P-cycling functions to specific microbial lineages. Yet existing genome-resolved studies remain largely confined to agricultural soils or individual ecosystems [11]. A key unresolved question is therefore howgenome-level P-cycling gene repertoires are shaped under chronic P scarcity and whether this organization can identify microbial lineages carrying P-mobilization genes.

Land use conversion reshapes soil structure and changes the energy and nutrients available to soil microorganisms [9, 12, 13]. Natural soils are generally less disturbed, whereas pasture soils receive sustained belowground carbon inputs from dense root systems and rhizodeposition [14]. Cropland soils, by contrast, are more disturbed microbial habitats, where tillage and inorganic fertilizer inputs repeatedly homogenize the soil environment [15]. These land use effects can act as environmental filters on soil P cycling by changing both the amount and chemical composition of soil P pools [16–18]. In Australian soils, conversion of native vegetation to cropping or pasture has been shown to alter total, organic, and inorganic P fractions, with particularly pronounced losses of organic P under long term cropping [19, 20]. Because different soil P pools are mobilized through distinct microbial pathways, shifts in soil P forms may further reshape microbial P-cycling strategies [21]. Yet whether and how land use conversion is associated with variation in these strategies at the continental scale remains poorly understood.

To resolve this gap, we combined metagenomic analyses of 268 soils across Australia with genome-resolved analysis of 1077 metagenome assembled genomes (MAGs). We focused on three major microbial P-cycling strategies: organic P mineralization, inorganic P solubilization, and P accumulation following the microbial phosphorus adaptive evolution framework [7]. These strategies regulate P mobilization, uptake, and storage in soil microbial communities under P-limited conditions. We hypothesize that (i) genes involved in P accumulation, organic P mineralization, and inorganic P solubilization show distinct distributions across Australian soils; (ii) genome-level repertoires are structured by phylogeny, with accumulation genes widely shared but mobilization genes lineage-restricted; and (iii) land use and soil properties are associated with variation in strategy-specific relative gene abundance. These analyses reveal the genome-level organization of microbial P-cycling strategies and their associations with land use and soil constraints in P-impoverished Australian soils.

## Materials and methods

### Soil sampling

Soil sampling was conducted across Australia’s 500–600 mm mean annual rainfall belt, where agricultural land use is widely represented. This design allowed us to examine how land use intensity is associated with soil phosphorus cycling under broadly comparable rainfall conditions. We established 65 sampling buffers along the 550 mm isohyet, with neighbouring buffers separated by ~100 km [22]. Each buffer was defined by a 30 km radius and adjusted where necessary to ensure that it fell primarily within the rainfall zone. Within each buffer, we targeted dominant pedogenic soil types and prioritized paired natural and agricultural sites. Paired sites were selected within 5 km of each other where possible, ensuring that land-use comparisons were made under similar soil type and soil-forming conditions. Topsoil was sampled from the 0–10 cm layer at each site, with three replicate subsamples taken within a 1 × 1 m quadrat. Because adjacent paired soils could not be identified at all locations, the final sampling design was adjusted accordingly. In total, 268 soil samples were collected across three Australian transects (Fig. S1), including Eastern Australia (EA; *n* = 142), Western Australia (WA; *n* = 71), and Northern Australia (NA; *n* = 55). These samples comprised natural soils (*n* = 107), cropland soils (*n* = 66), and pasture soils (*n* = 95). Specifically, natural soils were sampled under undisturbed native vegetation (forest, woodland, and bushland), pasture soils under grazed grassland, and cropland soils within cultivated fields growing wheat, canola, barley, alfalfa, or cotton. Sampling was conducted from April to November 2024. After collection, each sample was divided into three portions: ~20 g was frozen at −20°C for DNA extraction, ~500 g was air-dried and used for soil physicochemical analyses, and the remaining soil was kept at 4°C for biochemical measurements.

### Soil properties and climate gradients

Soil physicochemical properties were determined following established protocols [23]. Briefly, soil pH was determined using a calibrated pH meter after mixing soil with deionized water at a 1:5 ratio (w/v). Soil particle-size distribution was measured using the hydrometer method and reported as sand, silt, and clay fractions. Total carbon (TC) and total nitrogen (TN) were quantified by dry combustion using an elemental analyser. The soil C:N ratio was derived from the measured TC and TN concentrations. Total phosphorus (TP) was quantified after acid digestion by colorimetric determination following standard soil chemical procedures [23]. Particulate organic carbon and mineral-associated organic carbon (MAOC) were separated and quantified following published fractionation protocols [22, 24, 25].

Climatic data were obtained for each sampling coordinate using the ‘SLGACloud’ package in R [26, 27]. Extracted variables included mean annual temperature, mean annual precipitation, annual precipitation variability, annual atmospheric water deficit, and the aridity index.

### P-cycling gene annotation

Frozen soil subsamples (0.25 g) were used for microbial DNA extraction with the DNeasy PowerSoil Pro Kit (QIAGEN, USA), following the manufacturer’s instructions. After DNA quality assessment, the purified DNA was fragmented and prepared for sequencing using the ALFA-SEQ DNA Library Prep Kit (Findrop, China). Libraries were sequenced on a NovaSeq 6000 System (Illumina) to generate 150 bp paired-end reads, with ~20 Gbp of raw sequence data obtained for each sample. Raw reads were then processed using Trimmomatic v0.39 to remove adapters and low-quality sequences [28]. The resulting clean reads were assembled *de novo* with MEGAHIT v1.2.9 [29], and only contigs longer than 500 bp were retained to minimize the inclusion of short, potentially unreliable assemblies. Open reading frames (ORFs) were identified from the retained contigs using Prodigal v2.6.3 [30], and the predicted amino acid sequences were carried forward for functional annotation. Predicted proteins were searched against the PCycDB v1.1 protein database using DIAMOND blastp [31, 32]. For each query ORF, only the top hit was retained (-k 1) at an *E*-value threshold of 1 × 10^−5^. Putative phosphorus-cycling genes were further filtered using thresholds of ≥70% amino acid identity, ≥70% query coverage, and a minimum alignment length of 25 amino acids. The filtered ORFs were assigned to P-cycling gene families, generating an ORF-to-gene annotation table for each sample. To estimate the relative abundance of phosphorus-cycling genes, clean reads from each metagenome were mapped back to the predicted coding sequences (CDSs) using Salmon in metagenomic mode [33]. A sample-specific Salmon index was built from the predicted CDSs, and paired-end reads were quantified against this index. ORF-level relative abundances, expressed as transcripts per million (TPM), were extracted from the Salmon output and linked to the ORF-to-gene annotation table to obtain gene-level relative abundances.

### MAG reconstruction

For genome-resolved analysis, contigs shorter than 1000 bp were discarded, and the remaining contigs were processed with MetaWRAP v1.3.2 [34]. Bins were generated with the multiple binning algorithms implemented in MetaWRAP and consolidated with the MetaWRAP bin-refinement module to improve completeness and reduce contamination. The quality of the refined MAGs was assessed by CheckM2 v1.1.0 based on genome completeness and contamination estimates [35]. MAGs with completeness >50% and contamination <10% were retained following the MIMAG criteria for medium-quality draft genomes [36], allowing broad phylogenetic representation to be maintained in downstream analyses. To minimize redundancy among reconstructed genomes, retained MAGs were dereplicated with dRep v3.6.2 using the same quality thresholds [37]. This procedure generated 1077 dereplicated MAGs, including 979 bacterial MAGs and 98 archaeal MAGs. Their completeness and contamination metrics are reported in Table S1.

Taxonomic classification of the dereplicated MAGs was conducted with GTDB-Tk v.2.6.1 using GTDB release R226 [38]. To support phylogenetic interpretation of the reconstructed genomes, separate bacterial and archaeal phylogenomic trees were constructed from the GTDB-Tk concatenated marker-gene alignments. Bacterial MAGs were placed using the bac120 marker set, whereas archaeal MAGs were placed using the ar53 marker set. Both phylogenies were inferred using FastTree v2.2.0 [39] and were used to visualize the taxonomic distribution of phosphorus-cycling gene potential across the reconstructed MAGs. Protein-coding genes were predicted from each dereplicated MAG with Prodigal and annotated against PCycDB v1.1 following the same DIAMOND blastp search and filtering criteria described in the P-cycling Gene Annotation section, and filtered hits were mapped to PCycDB gene identifiers.

Due to the prevalence of P limitation in Australia, we focused on genes involved in microbial adaptive responses to phosphorus availability [7]. Given the importance of the balance between microbial phosphorus mineralization and immobilization in regulating phosphorus loss and ecosystem function [40], we focused our genome-resolved analysis on a set of established phosphorus-cycling marker genes: the mineralization genes (*phoA*, *phoD*, *phoX*, *phoN*), the solubilization gene (*gcd*), and the P accumulation genes, including phosphate uptake genes (*pstSCAB*) and the polyphosphate storage gene (*ppk*).

### Digital soil mapping of P-cycling genes

We mapped four P-cycling response variables across Australia: the combined relative abundance of P-cycling genes, and the relative abundances of P mineralizers, P solubilizers, and P accumulators to represent the key microbial functional groups governing soil phosphorus transformations. We modelled and mapped the relative abundance of each group using a digital soil biogeography framework [41–43]. In brief, gene relative abundances measured at the field sites were related to environmental predictors with random-forest models and projected across space on gridded covariate layers. For each response variable, a random-forest model of 250 trees was trained to predict gene abundance from 15 environmental covariates spanning climate, soil, terrain, and vegetation. The climate covariates were mean annual temperature, mean annual precipitation, and annual temperature range; the soil covariates were clay and sand content, available phosphorus, pH, soil organic carbon, and cation-exchange capacity; the terrain covariates were elevation and slope; and the vegetation covariate were seasonal Normalized Difference Vegetation Index values for four quarters. All covariates were obtained from the Soil and Landscape Grid of Australia via the ‘slga’ R package [44]. Soil covariates represented the 0–5 cm depth interval, and all covariate layers were aligned to a common 1 km grid. Model performance was evaluated across the 268 metagenomes using random 10-fold cross-validation, squared Pearson correlation and root mean square error. Final models were refitted using all samples and projected across Australia. We assessed environmental-range coverage as the proportion of covariates falling within their observed minimum–maximum ranges in the training samples [45, 46]. To limit extrapolation, we retained grid cells with at least 12 of the 15 environmental covariates (80%) within these ranges. This mask retained 59.9% of valid grid cells in the Australian prediction domain. All analyses were performed in R v4.3 using the ‘ranger’ [47] and ‘terra’ packages [48].

### Statistical analysis

Differences in relative P-cycling gene abundance among transects and among land-use types within each transect were tested using pairwise Wilcoxon rank-sum tests [49]. *P*-values for the comparisons were not adjusted for multiple testing, and borderline significant contrasts should therefore be interpreted cautiously. The variation in gene abundance associated with soil and climate was partitioned using redundancy analysis implemented in the ‘vegan’ package [50]. Predictor importance and effect direction were then modelled with gradient-boosted regression trees (XGBoost) interpreted with SHapley Additive exPlanations (SHAP) values [51]. Predictor importance was measured as the mean absolute SHAP value, and direction was measured by the Spearman correlation between a predictor and its SHAP value. Model performance was evaluated using five-fold cross-validated *R* [2]. Nonlinear responses of each group to its leading predictor were visualized with generalized additive models using the ‘mgcv’ package [52]. Genome-resolved analyses related MAG P-gene content to phylogeny and genome size using Mantel and partial Mantel tests [53]. All statistical analyses and visualizations were performed in R v4.3.

## Results

### Soil P-cycling gene repertoire across Australian soils

A total of 120 P-cycling gene families were identified across 268 Australian soil metagenomes using PCycDB annotation (Fig. 1a). At the process level, purine metabolism represented the largest component of the annotated repertoire, followed by two-component systems, transporters, and pyrimidine metabolism. Within the full repertoire, 43 gene families were detected in all samples, with mean relative abundance (TPM) values ranging from 3.9 to 41.1. The most abundant genes included the two-component response regulator *phoB*, the organic phosphate transporter *ugpC*, and nucleotide-associated genes such as *adk, nrdJ,* and *pyrG*. ORF-level taxonomic assignments further linked these P-cycling genes to a broad range of microbial phyla (Fig. S2). Bacterial lineages accounted for 90% of annotated P-cycling gene abundance, mainly represented by *Actinomycetota* (54%), *Pseudomonadota* (17%), and *Acidobacteriota* (8%). Archaea contributed only 6%, mostly from *Thermoproteota*.

**Figure 1:**
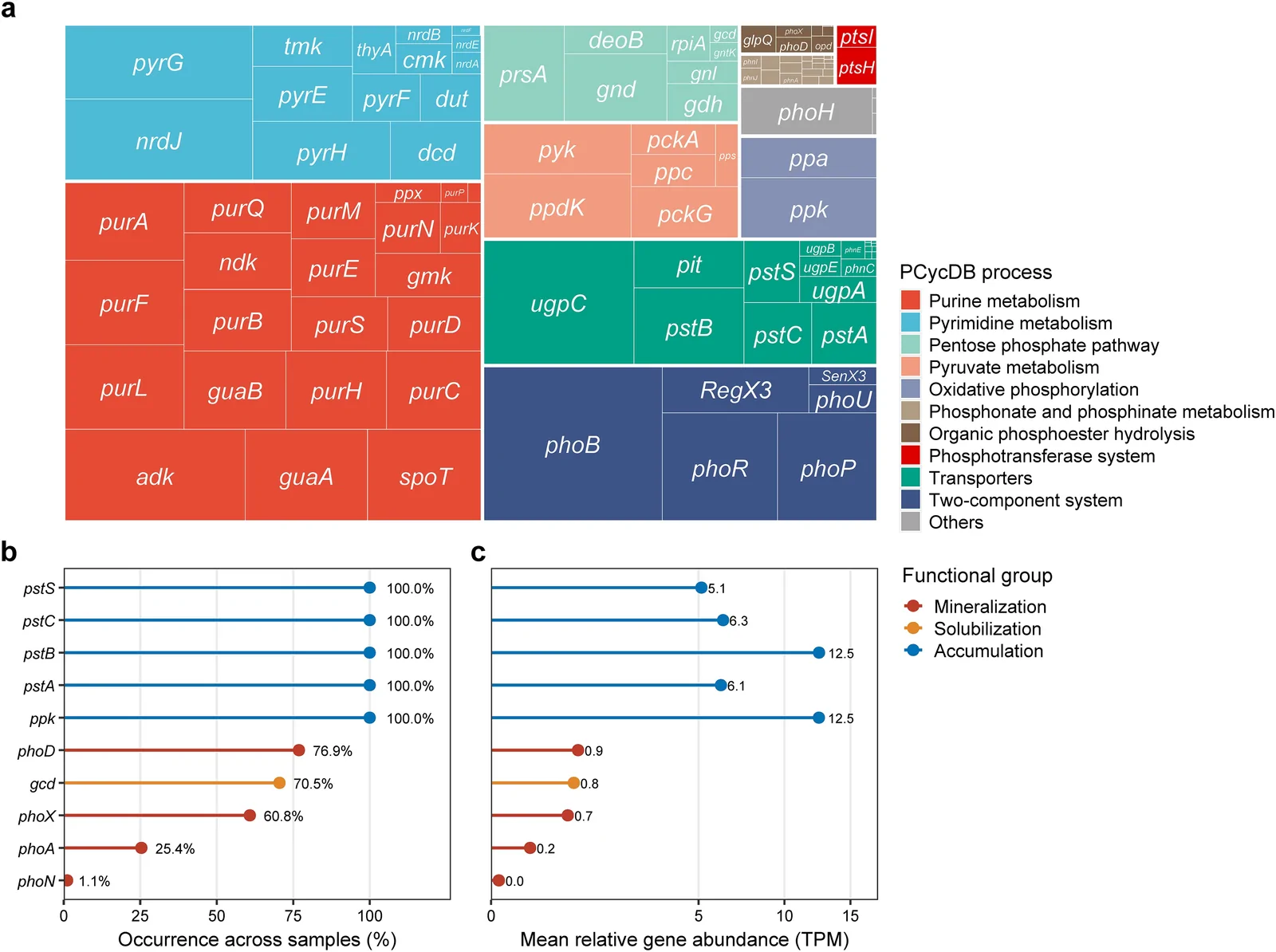
Relative abundance of PCycDB-annotated P-cycling genes. (a) Treemap showing the P cycling gene repertoire detected across all samples. Each rectangle represents one phosphorus cycling gene and rectangle area is proportional to the gene’s mean TPM across samples; genes are grouped and coloured by their assigned P cycling process. (b) Occurrence of the P-cycling genes. (c) Relative abundance (TPM) of different P-cycling genes across samples (square-root scale).

We then focused on genes directly related to microbial P acquisition and mobilization. These genes were grouped into three functional categories: organic-P mineralization (*phoA, phoD, phoX,* and *phoN*), inorganic-P solubilization (*gcd*), and phosphate accumulation (*pstS, pstC, pstA, pstB,* and *ppk*). The accumulation genes were detected in all samples and reached mean TPM values of 5.1–12.5 (Fig. 1b and c). By contrast, P mineralization genes showed lower and more variable prevalence, with *phoD, phoX, phoA*, and *phoN* detected in 76.9%, 60.8%, 25.4%, and 1.1% of samples, respectively. These genes also occurred at consistently low relative abundance, with mean TPM values below 1. The P solubilization gene *gcd* was detected in 70.5% of samples, with a mean TPM of 0.79.

### Genome-resolved distributions and phylogenetic structuring of P-cycling genes

From 268 metagenomes, we reconstructed 1077 medium- to high-quality metagenome-assembled genomes (MAGs), comprising 979 bacterial and 98 archaeal MAGs (Fig. S3a). All MAGs were classified at the phylum level, whereas only 5.0% of bacterial MAGs and 18.4% of archaeal MAGs could be assigned to a named species (Fig. S3b). Bacterial MAGs had higher GC content (mean 63.3% vs 37.7%) and larger genomes (mean 3.44 vs 1.93 Mb) than archaeal MAGs (Fig. S3c and d).

We then mapped the focal P-cycling genes onto phylogenomic trees of the bacterial and archaeal MAGs (Fig. 2). The bacterial MAGs spanned 20 phyla, dominated by *Actinomycetota, Acidobacteriota, Pseudomonadota,* and *Chloroflexota*, whereas all 98 archaeal MAGs belonged to a single phylum, *Thermoproteota* (Fig. 2a and c). The three functional categories differed in their distribution across bacterial phylogeny (Fig. 2a and b). Among the mineralization genes, the alkaline phosphatase gene *phoD* was the most widespread (42% overall, peaking at 68% in *Myxococcota*), whereas *phoA* was enriched in particular lineages, including *Verrucomicrobiota* (66%) and *Bacillota* (59%). The solubilization gene *gcd* occurred in 51% of MAGs but with a strong phylogenetic bias, reaching 100% in *Gemmatimonadota* and ≥ 80% in *Acidobacteriota, Vulcanimicrobiota,* and *Pseudomonadota*, yet falling to ~30% in the otherwise dominant *Actinomycetota* (29%) and *Chloroflexota* (31%). By contrast, phosphate uptake and storage genes were near-universal: the high-affinity phosphate-transporter gene *pstS* was present in 96% of MAGs and the polyphosphate kinase gene *ppk* in 80%.

**Figure 2:**
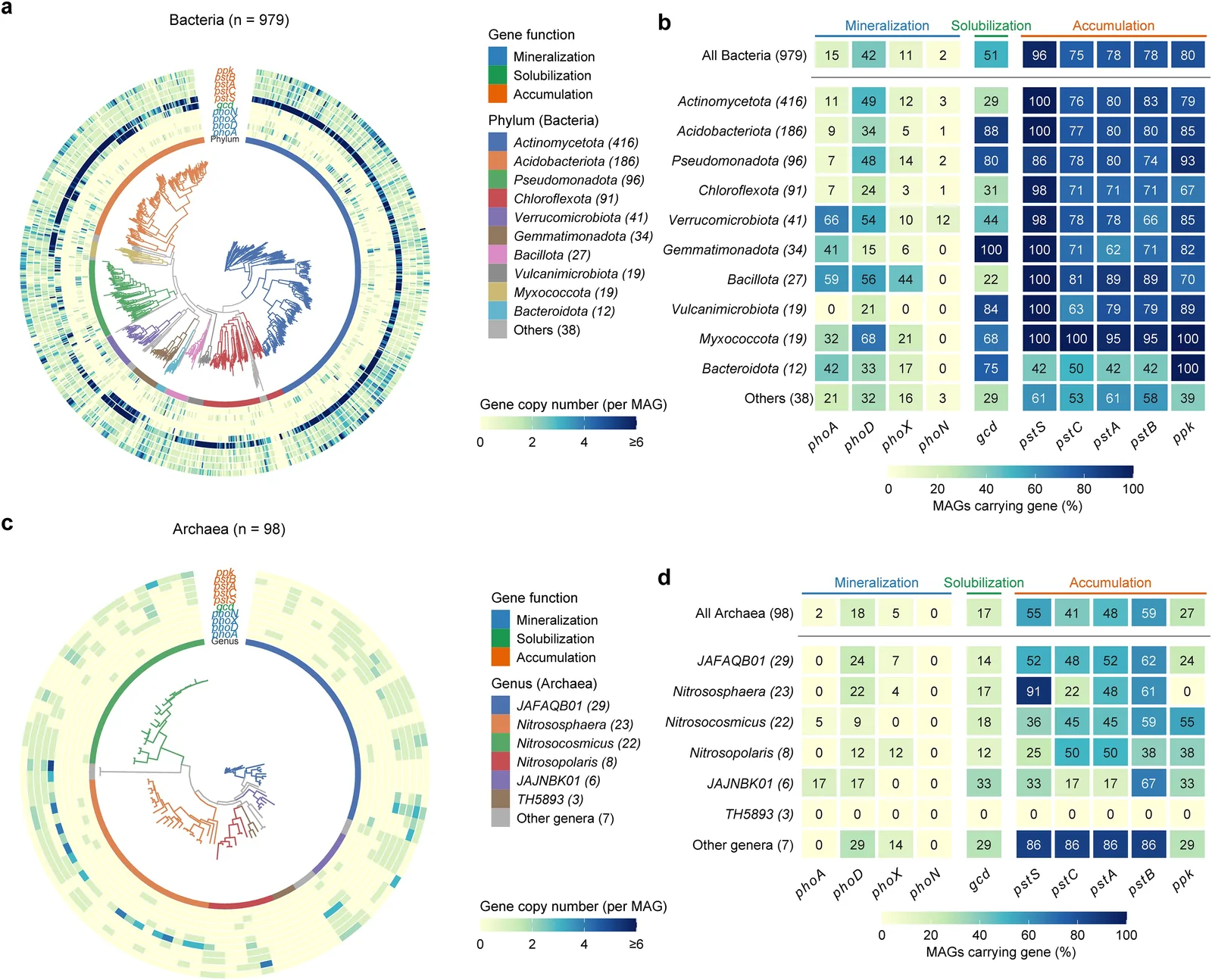
Phylogenetic distribution of phosphorus-cycling genes across bacterial and archaeal MAGs. (a) Phylogenomic tree of 979 bacterial MAGs, with branches coloured by the 10 most represented phyla and the remainder pooled as ‘Others’. Concentric rings show the per-MAG copy number of phosphorus-cycling genes, grouped by functional category: mineralization (*phoA*, *phoD*, *phoX*, *phoN*), solubilization (*gcd*) and accumulation (*pstS*, *pstC*, *pstA*, *pstB*, *ppk*); ring shading denotes gene copy number per MAG. (b) Prevalence of each gene. (c) Phylogenomic tree of 98 archaeal MAGs, with branches coloured by genus; rings as in (a). (d) Prevalence of each gene across archaeal genera with ≥3 MAGs (remaining genera pooled as ‘Other genera’).

To assess whether genome incompleteness inflated apparent gene absences, we repeated the analysis using 160 high-quality bacterial MAGs (>90% completeness and <5% contamination); the same broad pattern was retained, with the *pstSCAB* components and *ppk* occurring more frequently than most mineralization and solubilization genes (Fig. S4). However, this high-quality subset mainly comprised *Actinomycetota*, *Acidobacteriota*, *Pseudomonadota,* and *Chloroflexota*, with the remaining phyla represented by relatively few high-quality MAGs and therefore pooled as ‘Other phyla’ rather than evaluated separately.

In the archaeal MAGs (all *Thermoproteota*), the same categories showed a narrower, transport-centred pattern (Fig. 2c and d). Mineralization was limited and confined almost entirely to *phoD* (18% overall), with *phoA, phoX,* and *phoN* each detected in ≤5% of MAGs. Solubilization via *gcd* was similarly sparse (17%). Phosphate acquisition dominated the archaeal repertoire, with the *pst* transporter genes encoded by 41%–59% of MAGs, whereas storage was comparatively rare: *ppk* occurred in only 27% of MAGs and was absent from several genera. This pattern was evident in *Nitrososphaera*, which showed the highest transporter prevalence (*pstS* 91%) but lacked *ppk*.

To examine the factors associated with the uneven distribution of P-cycling genes, we focused on the 979 bacterial MAGs. The number of P-cycling genes per MAG increased with genome size (Fig. 3a). This relationship was function-specific: accumulation genes were nearly universal even in the smallest genomes, whereas mineralization and solubilization genes were more frequently detected in larger genomes (Fig. 3b). This genome-size effect was also observed within phyla, with most phyla showing a similar positive association between genome size and P-cycling gene richness (Fig. S5).

**Figure 3:**
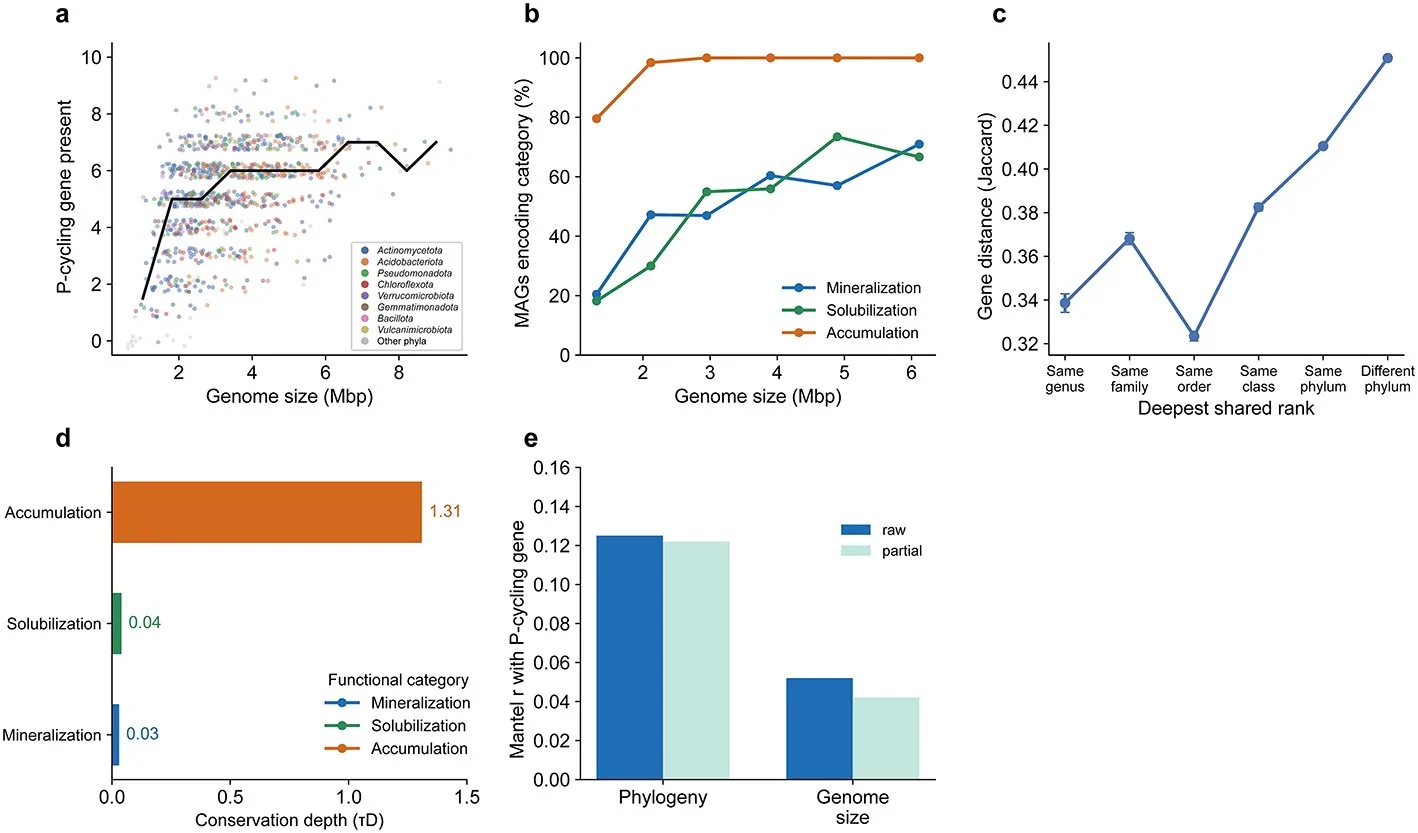
Phosphorus-cycling gene content in bacterial MAGs is shaped more by phylogeny than by genome size. (a) Number of different P-cycling genes present per MAG versus genome size. Each point represents a bacterial MAG, with phylum assignments indicated in the legend; the solid line represents the median within each genome-size bin. (b) Percentage of MAGs encoding each functional category (mineralization, solubilization, accumulation) across genome-size bins. (c) Mean difference in P-gene content between pairs of MAGs, grouped by the deepest taxonomic rank they share (same genus → different phylum). (d) Phylogenetic conservation depth of each category. (e) Mantel tests relating between-MAG differences in P-cycling genes content to phylogenetic distance and genome-size difference. Bars represents Mantel *r* value.

Because genome size often covaries with phylogenetic lineage, we next tested whether bacterial P-cycling repertoires were more strongly associated with genome size or phylogeny. P-cycling gene composition was strongly phylogenetically structured, with gene-content dissimilarity increasing with phylogenetic distance (Fig. 3c). All three functional categories showed phylogenetic conservation, but their conservation depths differed: accumulation genes were conserved across deeper nodes, whereas mineralization and solubilization genes were restricted to more lineage-specific clades (Fig. 3d). Mantel tests further showed that P-gene composition was more strongly associated with phylogenetic distance than with genome-size differences (Fig. 3e). Beyond this lineage structure, the three functional categories showed weak co-occurrence across genomes. The only strong gene-level co-occurrence occurred within the *pst* transport operon (Fig. S6a), which formed a conserved and physically linked gene cluster across different MAGs (Fig. S6b).

### Land-use variation in focal P-cycling functions

Although the combined relative abundance of P-cycling genes showed only a weak relationship with geographic distance (Fig. S7), it differed significantly among transects. The total relative abundance of P-cycling genes and all three functional categories was highest in WA, intermediate in EA, and lowest in NA (Fig. 4a; *P* < .05). Land-use-associated differences were evaluated within EA and WA only because samples in NA were all from pasture soils and did not allow within-transect land-use comparisons.

**Figure 4:**
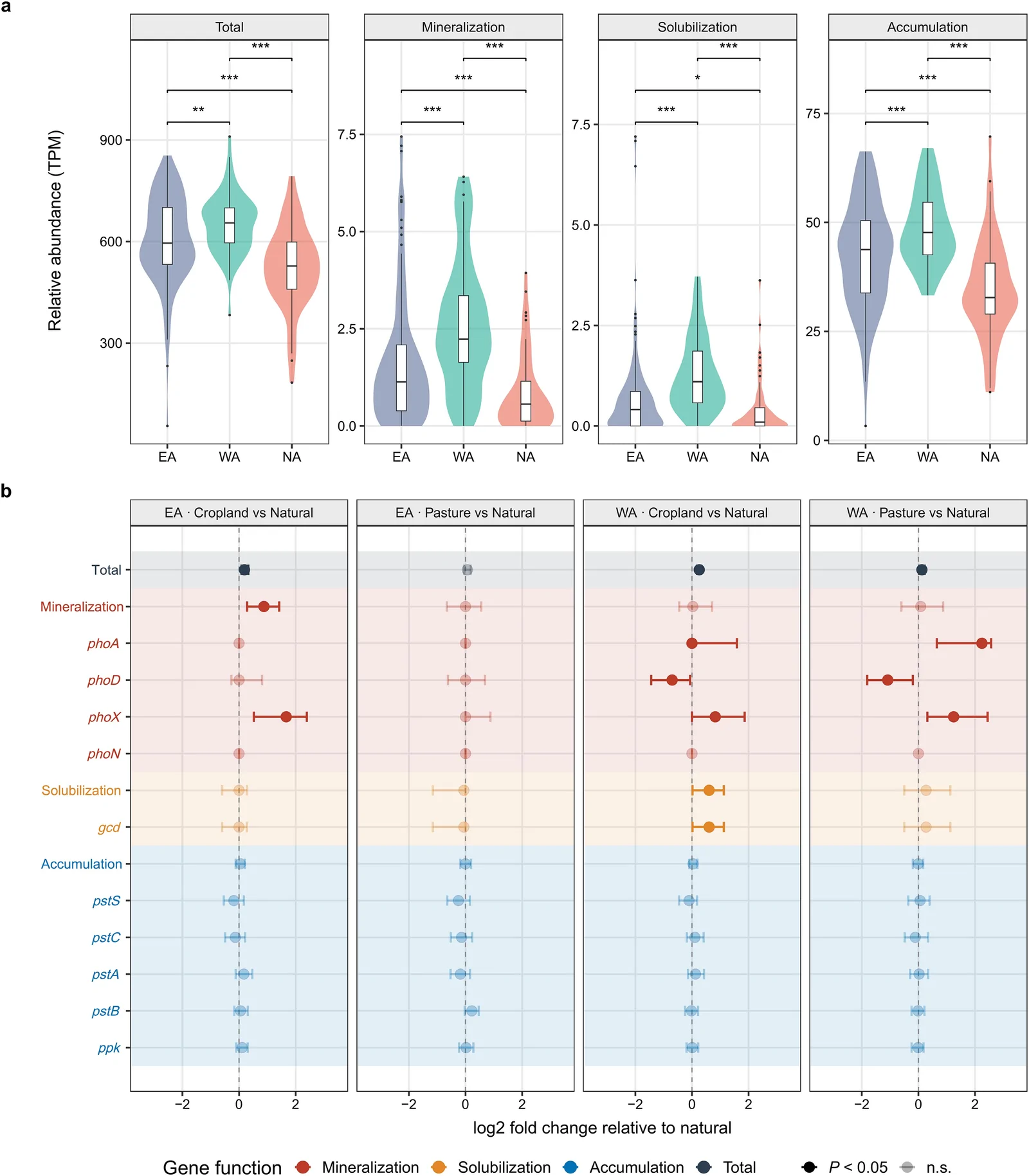
Geographic and land-use influence on P-cycling genes across Australian soils. (a) Abundance (TPM) of P-cycling genes across the three transects. Horizontal brackets denote pairwise Wilcoxon rank-sum tests between transects (^*^P < .05, ^**^P < .01, ^***^P < .001). (b) Log₂ fold-change in gene abundance under cropland or pasture relative to natural vegetation, within the EA and WA transects. EA, eastern Australia, (*n* = 142); WA, western Australia, (*n* = 71); NA, northern Australia, (*n* = 55).

Land-use-associated patterns differed between regions (Fig. 4b). Cropland soils in EA were associated with a 14% higher total relative abundance of P-cycling genes, whereas pasture showed no significant association with this metric. In WA, cropland and pasture showed broadly similar patterns. Both land uses were associated with higher total relative abundance of P-cycling genes, by 19% under cropland and 10% under pasture. Although neither land use was significantly associated with the total relative abundance of mineralization genes in WA, both cropland and pasture were associated with higher relative abundances of *phoA* and *phoX* but lower *phoD* abundance. In contrast, accumulation genes showed no significant land-use-associated differences in either EA or WA. A complementary analysis of land-use sites paired within 5 km showed higher total relative abundance of P-cycling genes in cropland than in matched natural soils, whereas *gcd* abundance was lower in pasture. Accumulation genes showed no significant differences in either paired comparison (Fig. S8). These land-use contrasts also occurred against distinct edaphic backgrounds, with significant land-use differences in pH, C:N ratio, total P, and fMAOC depending on the transect (Fig. S9). Total P was higher in cropland than in natural and pasture soils in both EA and WA.

### Edaphic controls dominate over climate in explaining P-cycling gene abundance

Variation partitioning identified soil properties as the dominant environmental component explaining P-cycling gene abundance (Fig. 5a). Soil properties accounted for the largest unique fraction of variation in total P-cycling genes, accumulation genes, and mineralization genes, explaining 32%, 16%, and 18% of the variation, respectively. Consistent with this edaphic dominance, the XGBoost–SHAP analysis identified different soil predictors for different P-cycling functional groups (Fig. 5b). Soil pH was the leading predictor for total P-cycling and mineralization genes. Total P-cycling gene abundance showed a unimodal response to pH, peaking in mildly acidic soils around pH 5–6 (Fig. 5c), whereas mineralization genes increased with pH before reaching a plateau (Fig. 5d). Clay content was the leading predictor for solubilization genes, which declined with increasing clay content (Fig. 5e). fMAOC was the leading predictor for accumulation genes, which decreased sharply when fMAOC exceeded ~0.6 (Fig. 5f). At the individual-gene level, response curves further supported this pattern, with most genes being associated primarily with soil physicochemical predictors rather than climatic variables (Fig. S10).

**Figure 5:**
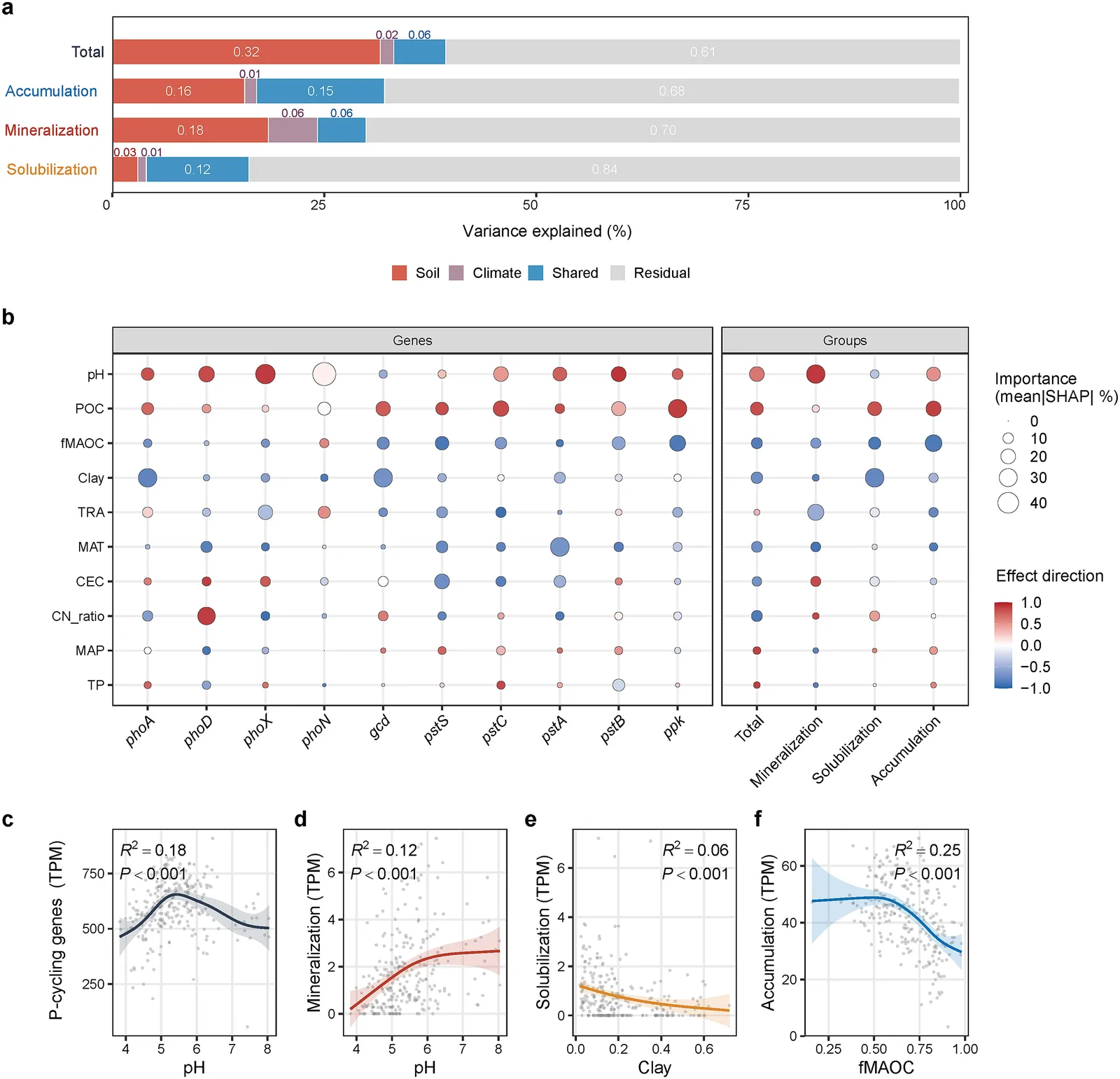
Environmental drivers of soil microbial phosphorus (P)-cycling potential. (a) Variation partitioning of P-cycling gene abundance into unique and shared fractions explained by soil properties and climate. (b) Importance and direction of environmental predictors for individual P-cycling genes and functional groups, estimated using gradient boosted regression (XGBoost) combined with SHapley Additive exPlanations (SHAP). Point size indicates relative importance, calculated from mean absolute SHAP values, and colour indicates the correlation direction between each predictor and its SHAP value. (c–f) Fitted response curves for each functional group against its leading environmental predictor: (c) total P-cycling genes; (d) mineralization genes; (e), solubilization genes; and (f), accumulation genes. MAP, mean annual precipitation; MAT, mean annual temperature; TRA, annual temperature range; CEC, cation exchange capacity; TP, total phosphorus; POC, particulate organic carbon; fMAOC, the fraction of MAOC in total organic carbon; C:N ratio, soil carbon-to-nitrogen ratio.

### Continental predictions reveal spatially structured P-cycling genes

Random-forest models were used to project the relative abundance of P-cycling genes using soil, climate, terrain, and vegetation variables (Fig. 6). The results showed that the combined relative abundance of P-cycling genes was generally higher in south-western and coastal eastern Australia (Fig. 6a). P-solubilization and P-accumulation genes showed broadly similar coastal patterns (Fig. 6c and d), whereas P-mineralization genes showed relatively higher predicted values in southern Australia (Fig. 6b). Cross-validation performance differed among functional groups (Fig. S11), with the highest *R*^2^ for combined P-cycling genes (*R*^2^ = 0.61), followed by P accumulation (*R*^2^ = 0.32), P mineralization (*R*^2^ = 0.19), and P solubilization (*R*^2^ = 0.05). The category-specific projections, particularly those for mineralization and solubilization, should be interpreted as broad spatial trends. Predictor importance also varied among functional groups (Fig. S12).

**Figure 6:**
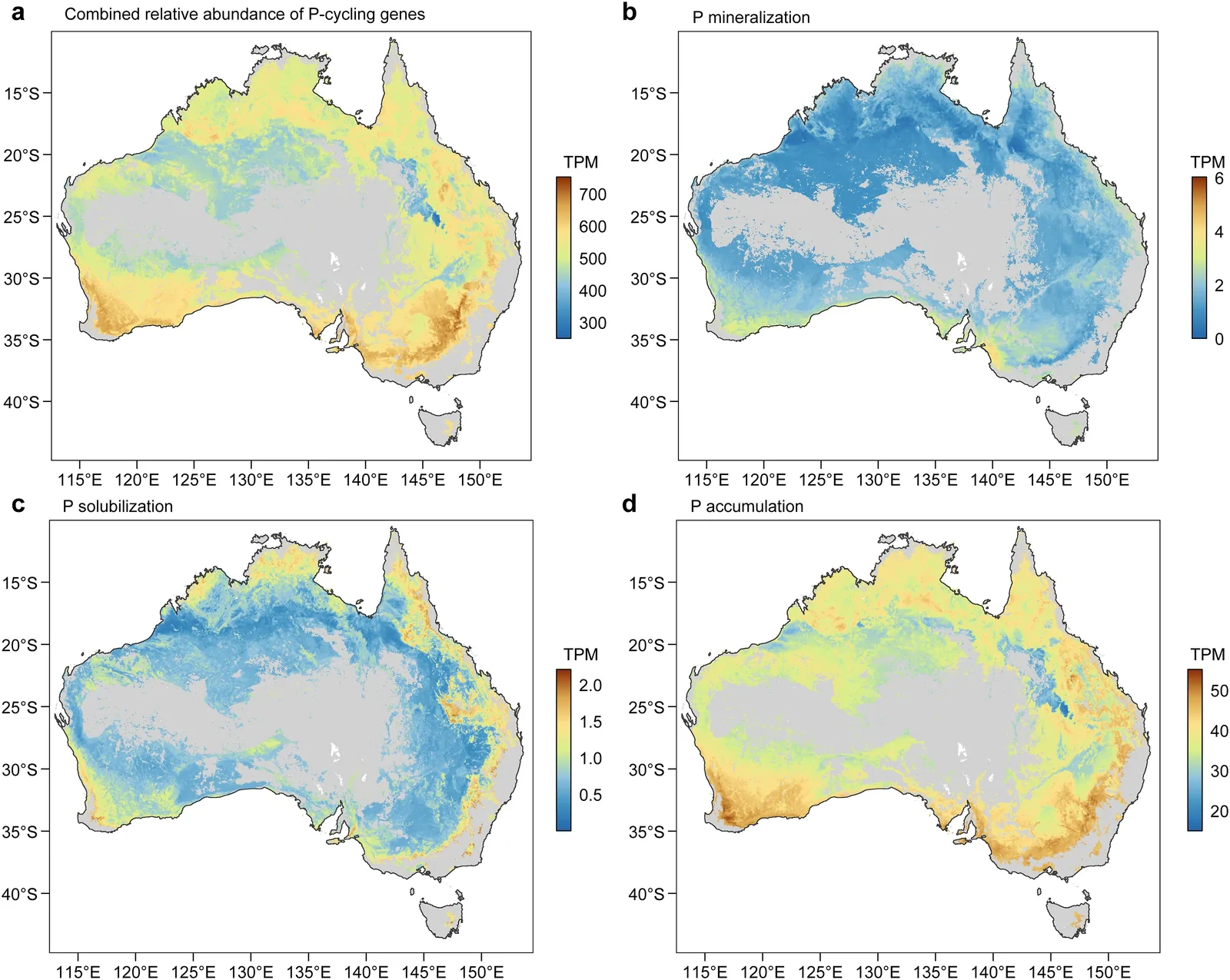
Predicted spatial distributions of the relative abundance of soil phosphorus-cycling genes across Australia. (a) Combined relative abundance of PCycDB-annotated P-cycling genes; (b) P-mineralization genes (*phoA, phoD, phoX,* and *phoN*); (c) the P-solubilization gene *gcd*; and (d) P-accumulation genes (*pstA, pstB, pstC, pstS,* and *ppk*). Values represent summed TPM-normalized relative gene abundances within each category. Grey areas indicate locations with limited environmental representation in the training data and are excluded from spatial interpretation. Panel c shows low-confidence predictions for P-solubilization (*R*^2^ = 0.05), which should be interpreted cautiously.

## Discussion

### Contrasting genomic distributions of soil phosphorus-cycling strategies

Our genome-resolved analysis reveals a clear continental pattern in microbial P cycling. Genes involved in phosphate accumulation were widespread across bacterial genomes, whereas genes involved in P mobilization were concentrated in specific lineages. The broad distribution of accumulation genes is consistent with their core physiological roles. The *pstSCAB* system encodes a high-affinity phosphate transporter that imports inorganic phosphate into cells, whereas *ppk* encodes a polyphosphate kinase involved in polyphosphate synthesis and storage [54]. These functions support phosphate homeostasis and intracellular P management, which may explain the high occurrence of *pstSCAB* and *ppk* across bacterial MAGs in chronically P-impoverished soils [55]. By contrast, genes associated with P mobilization showed stronger lineage specificity. The *gcd* gene encodes membrane-bound quinoprotein glucose dehydrogenase and is commonly used as a marker for mineral phosphate solubilization [56]. In our MAGs, *gcd* was enriched in several phyla not traditionally regarded as phosphate solubilizers such as *Acidobacteriota, Gemmatimonadota, Myxococcota,* and *Bacteroidota*. This extends previous genome-resolved evidence that *gcd*-carrying genomes occur outside classical phosphate-solubilizing groups [11, 56]. Organic P mineralization genes showed a similarly restricted distribution. *phoD* was the most prevalent phosphatase-encoding gene, whereas *phoA, phoX,* and *phoN* occurred in fewer recovered MAGs. This uneven distribution suggests that alternative phosphatase pathways are not equally accessible across soil microbial lineages [57].

### Land use is more strongly associated with P-mobilization than P-accumulation pathways

Land use was more strongly associated with variation in P-mobilization genes than in phosphate accumulation genes. In the transect where land-use contrasts were available, cropland generally showed a higher relative abundance of total P-cycling genes than natural soils. This pattern is consistent with global evidence that low-to-moderate human intervention can increase soil P-cycling gene abundance [10]. The increase also coincided with higher soil total P under cropland, suggesting that fertilizer inputs and altered P pools may enrich microbial groups carrying P-mobilization pathways [16]. Higher soil P under cropland may reflect altered P availability and microbial P demand, potentially contributing to shifts in communities carrying P-mobilization pathways [58, 59]. However, land-use-associated variation was not uniform among mineralization genes. Cropland was associated with higher *phoX* abundance, whereas *phoD* declined only in WA. The increase in *phoX* may be partly due to cropland-associated changes in soil pH because *phoX* encodes an alkaline phosphatase and *phoX*-harbouring communities are strongly shaped by soil pH and land use [60]. In addition, the positive association between *phoX*-harbouring communities and soil total P further supports the link between cropland P enrichment and *phoX*-based mineralization potential [61]. By contrast, the decline of *phoD* in WA may indicate reduced reliance on *phoD*-mediated organic P mineralization, or a shift towards microbial lineages carrying alternative phosphatase pathways [60]. In contrast to mobilization genes, phosphate accumulation genes showed little response to land-use conversion. Unlike the extracellular mineralization genes, *pstSCAB* and *ppk* mediate cellular P uptake and polyphosphate storage and may respond to P availability through regulation and polyphosphate turnover rather than through changes in gene abundance [59].

### Edaphic conditions explain strategy-specific variation in P-cycling genes

Local edaphic conditions were more strongly associated with variation in microbial P-cycling genes than broad climatic gradients. Although recent studies have described the global distribution of soil P-cycling microbes, the environmental associations of strategy-specific P-cycling genes remain poorly resolved [10]. Here, soil properties explained more variation in the relative abundance of microbial P-cycling genes than climatic variables, suggesting that local edaphic conditions are closely related to microbial P acquisition strategies. Soil pH emerged as the strongest predictor of the relative abundance of PCycDB-annotated P-cycling genes and mineralization genes. This is consistent with the central role of pH in structuring soil bacterial communities at continental scales [62]. However, whether pH also shapes P-cycling genetic potential in a strategy-specific manner remains less clear. In our study, total P-cycling genes peaked in mildly acidic soils, around pH 5–6. This pattern may indicate a balance between P acquisition demand and microbial pH tolerance because strong acidification can constrain microbial P-solubilizing and P-mineralizing capacity [59]. Soil texture was also associated with variation in P-cycling strategies, particularly mineral P solubilization. Clay minerals are important P-binding constituents and can adsorb inorganic phosphate onto mineral surfaces [63]. Therefore, the decline of solubilization genes with increasing clay content may indicate reduced accessibility of mineral-bound P in clay-rich soils. This suggests that soil texture can influence not only P retention but also the microbial genetic potential required to mobilize mineral-associated P.

The negative association between accumulation genes and fMAOC points to a potential link between microbial P management and soil organic matter stabilization. Although soil total phosphorus is often positively associated with soil organic matter, the role of microbial P-cycling genes in carbon persistence remains unclear [64]. This gap is important because phosphorus dynamics can regulate mineral-associated organic matter (MAOM) formation and persistence [65]. In our study, accumulation genes mainly included *pstSCAB* and *ppk*, representing inorganic phosphate uptake and intracellular polyphosphate storage potential [66]. Their decline with increasing fMAOC may indicate lower growth-related P uptake and storage demand in soils dominated by mineral-associated carbon. One possible explanation is that high fMAOC means a lower contribution of particulate or labile organic carbon, which can reduce microbial activity hotspots and nutrient demand [67, 68]. This pattern suggests that microbial P-acquisition traits should be considered when linking phosphorus cycling to belowground carbon persistence.

### Continental prediction identifies regional patterns in P-cycling gene abundance

Our spatial predictions identify wetter eastern coastal and southern regions with higher relative abundance of microbial P-cycling genes across Australia. The model for total P-cycling genes performed best, indicating that broad P-cycling potential has a strong environmental signal. By contrast, predictive performance was weaker for the three individual functional categories. We therefore treat the predicted map of combined P-cycling gene abundance as the main spatial prediction, whereas the category-specific maps are used mainly to compare broad differences among P-cycling strategies. Solubilization genes were predicted least well. This weaker predictability may be due to the biology of *gcd*, whose host microbes thrive across a wide pH range [69], and span diverse taxa [70]. Solubilization may also depend on mineral-surface properties, organic-acid production, and local pH conditions, which are not fully captured by continental soil and climate layers [71, 72]. Our continental predictions also differed from previous global projections of soil P-cycling microbes [10]. This discrepancy may reflect differences in sampling density, spatial scale, and model structure. Australia is a highly weathered and chronically P-impoverished continent, so our observations can resolve within-continent variation in microbial P-cycling potential. These maps therefore provide a spatial baseline for identifying broad regional patterns in microbial P-cycling gene distributions.

Overall, our continental survey addresses an important gap in the biogeography of soil P-cycling genes across Australia. We show that chronically P-impoverished soils harbour diverse microbial P-cycling gene repertoires and that their relative abundances are associated with land use and local edaphic conditions. Our genome-resolved analysis further reveals contrasting genomic distributions among microbial P-cycling strategies, with phosphate accumulation genes being widely distributed and P-mobilization genes being more phylogenetically constrained. By linking community-level gene abundance, MAG-level repertoires, and spatial predictions, this study provides a framework for understanding where microbial P-cycling potential occurs, which lineages carry key P strategies, and how these strategies respond to land-use and soil constraints. However, several limitations remain. First, TPM-based gene abundances represent relative functional potential rather than absolute abundance or realized activity; independent qPCR validation would be required to determine whether these relative patterns correspond to differences in absolute gene abundance. Second, land-use-associated differences were inferred from observational contrasts, so the causal links among land use, soil P forms, and P-cycling gene abundance require experimental testing. Third, continental predictions were constrained by uneven spatial and environmental representation, particularly in inland Australia, and should therefore be interpreted as broad regional patterns. Finally, the negative association between P-accumulation genes and fMAOC is correlative, and the mechanisms coupling microbial P acquisition with the partitioning of soil organic carbon into mineral-associated and particulate pools require further investigation. Despite these limitations, our findings provide a genome-resolved and spatially explicit basis for understanding the distribution and environmental associations of microbial P-cycling genes in chronically P-impoverished soils.

## Supplementary Material

Supplementary_materials_wrag272

## Data Availability

The datasets generated during and/or analysed during the current study are available in the Zenodo repository, doi:10.5281/zenodo.23113556.
