# Supplementary material for: Contrasting edaphic and land-use signatures of microbial phosphorus mobilization and accumulation genes
Source: ISME J. 2026 Oct 20;20(1):wrag272. doi: 10.1093/ismejo/wrag272 (PMC13768749; doi:10.1093/ismejo/wrag272)
Supplement: Supplementary file 1 [file supplementary_materials_wrag272.docx]

Supplementary materials:

Fig. S1. Sampling locations. Map showing the geographic distribution of 268 soil metagenome samples collected across three Australian transects: Eastern Australia (n = 142), Western Australia (n = 71), and Northern Australia (n = 55).

Fig. S2. Taxonomic distribution of P-cycling-carrying ORFs across microbial phyla. Bubble plot showing the distribution of P-cycling genes across phylum-level host assignments. Columns represent P-cycling gene families grouped by process-level annotation, and rows represent bacterial, archaeal and unclassified host assignments. Point size indicates mean TPM assigned to each gene-phylum combination, and colour indicates the mean relative abundance.

Fig. S3. Composition, quality, taxonomic novelty and genomic features of the recovered MAGs. (a) Domain composition of the 1,077 MAGs (979 Bacteria, 98 Archaea). (b) GTDB-Tk classification depth: percentage of MAGs assigned at each rank for Bacteria (blue) and Archaea (orange). (c) GC content and (d) genome-size distributions, shown as density histograms with Gaussian kernel-density estimates (dashed lines, domain means).

Fig.S4: Prevalence of P-cycling genes across 160 high-quality bacterial MAGs (completeness >90% and contamination <5%), shown overall and for major phyla, with cell colours and values representing the percentage of MAGs containing each gene.

Fig. S5. The genome-size effect on P-cycling categories within bacterial phyla.
Prevalence (% of MAGs encoding ≥1 gene) of mineralization, solubilization and acquisition/accumulation across genome-size tertiles within each bacterial phylum represented by ≥25 MAGs.

Fig. S6. Modularity of phosphorus-cycling functions across bacterial MAGs.
(a) Genome-level co-occurrence (φ coefficient on gene presence/absence) among the 10 focal P-cycling genes across 979 bacterial MAGs, coloured by functional category; the top bar shows each gene's prevalence. (b) Genomic context of the *pst* operon in a representative MAG from each of six bacterial phyla, reconstructed from Prodigal gene coordinates and aligned at the *pst* operon start. Genes are coloured by function and arrows indicate transcriptional strand (flanking non-P genes in grey)

Fig.S7. Distance-decay of soil phosphorus-cycling gene composition across Australia.

Fig.S8. Paired land-use-associated differences in relative P-cycling gene abundance. Points represent mean paired log₂ fold-changes relative to natural soils, and horizontal bars indicate 95% confidence intervals. P values were obtained using two-sided paired t-tests on log₂-transformed abundances.

Fig.S9. Soil properties under different land use in different transect.

Fig.S10. Each P-cycling marker gene versus its strongest environmental driver.
Abundance (TPM) of each of the ten P-cycling marker genes (gene name above each panel) plotted against the environmental predictor with the highest SHAP importance for that gene, shown as GAM smooths with 95% confidence bands; the adjusted *R*² and the smooth-term *P* value are given in each panel. Colours denote functional group (red, mineralization; amber, solubilization; blue, accumulation). *n* = 261 soil samples.

Fig.S11. Ten-fold Cross-validation performance of the random-forest models used for digital soil mapping. Observed versus ten-fold cross-validated predictions of gene abundance for each P-cycling group. The dashed line shows perfect agreement (predicted = observed); the solid red line shows the linear fit to the data. R² and RMSE are from the cross-validation (n = 268).

Fig. S12. Environmental drivers of P-cycling gene relative abundance in the random-forest models used for digital soil mapping. (a) Impurity-based random-forest variable importance (relative %) of 15 covariates for each group. (b–e) Partial-dependence plots of each group's top predictor: (b) total–MAT, (c) mineralizers–TRA, (d) solubilizers–slope, (e) accumulators–MAT. Rug marks show the data distribution. MAT, mean annual temperature; MAP, mean annual precipitation; TRA, temperature annual range; SOC, soil organic carbon; CEC, cation exchange capacity; P, available phosphorus; NDVI_Q1–Q4, quarterly NDVI (Normalized Difference Vegetation Index)

Table S1**.** Completeness and contamination of bacterial and archaeal MAGs included in Figure 2.

| **Taxonomic group** | **n** | **Completeness (%)** | **Contamination (%)** |
| --- | --- | --- | --- |
| **Bacteria: phylum-level groups** | | | |
| *Actinomycetota* | 416 | 74.04 ± 14.25 | 3.38 ± 2.36 |
| *Acidobacteriota* | 186 | 75.53 ± 16.16 | 3.43 ± 2.25 |
| *Pseudomonadota* | 96 | 69.47 ± 13.98 | 3.43 ± 2.30 |
| *Chloroflexota* | 91 | 69.37 ± 14.67 | 4.03 ± 2.26 |
| *Verrucomicrobiota* | 41 | 71.00 ± 14.02 | 3.28 ± 2.18 |
| *Gemmatimonadota* | 34 | 69.86 ± 13.40 | 3.59 ± 2.73 |
| *Bacillota* | 27 | 66.85 ± 11.17 | 3.76 ± 2.21 |
| *Vulcanimicrobiota* | 19 | 68.34 ± 15.90 | 3.44 ± 2.30 |
| *Myxococcota* | 19 | 74.06 ± 14.27 | 3.89 ± 2.32 |
| *Bacteroidota* | 12 | 69.11 ± 17.49 | 2.76 ± 1.60 |
| Other | 38 | 75.46 ± 14.89 | 3.79 ± 3.05 |
| **Archaea: genus-level groups** | | | |
| *JAFAQB01* | 29 | 73.84 ± 16.93 | 3.49 ± 2.17 |
| *Nitrososphaera* | 23 | 71.42 ± 14.51 | 2.35 ± 2.06 |
| *Nitrosocosmicus* | 22 | 66.13 ± 13.42 | 3.02 ± 2.65 |
| *Nitrosopolaris* | 8 | 73.75 ± 13.51 | 3.00 ± 2.72 |
| *JAJNBK01* | 6 | 64.29 ± 17.06 | 4.46 ± 2.55 |
| *TH5893* | 3 | 54.40 ± 3.81 | 3.79 ± 3.25 |
| Other genera | 7 | 76.31 ± 20.64 | 2.07 ± 2.06 |

**Note:** Values are mean ± SD. Taxonomic groups and sample sizes correspond exactly to those shown in Figure 2. Other includes 38 bacterial MAGs assigned to phyla not shown separately; Other genera include seven archaeal MAGs assigned to genera not shown separately.
